# High-resolution Identification and Separation of Living Cell Types by Multiple microRNA-responsive Synthetic mRNAs

**DOI:** 10.1038/srep21991

**Published:** 2016-02-23

**Authors:** Kei Endo, Karin Hayashi, Hirohide Saito

**Affiliations:** 1 Department of Life Science Frontiers, Center for iPS Cell Research and Application, Kyoto University, 53 Kawahara-cho, Shogoin, Sakyo-ku, Kyoto 606-8507, Japan

## Abstract

The precise identification and separation of living cell types is critical to both study cell function and prepare cells for medical applications. However, intracellular information to distinguish live cells remains largely inaccessible. Here, we develop a method for high-resolution identification and separation of cell types by quantifying multiple microRNA (miRNA) activities in live cell populations. We found that a set of miRNA-responsive, *in vitro* synthesized mRNAs identify a specific cell population as a sharp peak and clearly separate different cell types based on less than two-fold differences in miRNA activities. Increasing the number of miRNA-responsive mRNAs enhanced the capability for cell identification and separation, as we precisely and simultaneously distinguished different cell types with similar miRNA profiles. In addition, the set of synthetic mRNAs separated HeLa cells into subgroups, uncovering heterogeneity of the cells and the level of resolution achievable. Our method could identify target live cells and improve the efficiency of cell purification from heterogeneous populations.

Multicellular organisms consist of many cell types, each with different function^1^. The identification and separation of target living cells is critical not only to reveal their function, but also to use them for therapeutic purposes. Marker proteins on the cell surface are commonly used to identify living cell types^2^,^3^,^4^,^5^. However, obtaining specific sets of surface markers and their corresponding antibodies is not trivial. Moreover, these antibody-detected cell populations often remain heterogeneous and composed of subgroups that cannot be distinguished to each other^6^. To improve the resolution of live cell identification and separation, means for quantitative detection and utilization of intracellular markers are essential.

MicroRNAs (miRNAs), which are short non-coding RNAs transcribed in plants and animals^7^,^8^ are a potential non-protein intracellular biomarker to distinguish cell types^9^,^10^. The nature of nucleic acids is suitable for quantitative measurement in a high-throughput manner^11^,^12^,^13^ despite difficulty in applying such methods to live cells. The activity of miRNA is controlled during various biological processes^14^. Thus, the measurement of miRNA activity, rather than that of miRNA quantity, is more suitable for live cell identification and separation. We have recently reported a method to purify cell populations using synthetic mRNA that detects highly expressed miRNA in target cells^15^. However, identification and separation of cells with similar miRNA profiles remains a challenge, though it is particularly important for the quality control of cells in therapeutic applications.

In this study, we developed a means for the high-resolution identification of cell types (HRIC) based on the simultaneous quantification of multiple miRNA activities in live cells using a set of miRNA-responsive, synthetic mRNAs. Transfection of two *in vitro* synthesized mRNAs that encode different fluorescent proteins (FL1 or FL2) enabled us to detect a specific cell population as a peak (Supplementary Fig. S1), because the fluorescence ratio of the reporter proteins from the two mRNAs (FL2/FL1) was almost constant irrespective of the transferred mRNA levels in each cell. A single miRNA target site, which is completely complementary to the target miRNA, in the 5′ UTR of synthetic mRNAs efficiently detected miRNA activity in a cell^15^. Then, we assumed that the combination of two distinct miRNA-responsive mRNAs can improve the resolution for cell identification: If two miRNAs are more and less active, respectively, in one cell type compared with the other cell type, then subtle differences in miRNA activities should be sufficient to distinguish them (Fig. 1). Because we found that the peak width of the fluorescence ratios translated from two reporter mRNAs tends to distribute within four-fold, using our strategy, less than two-fold differences in two miRNA activities, which result in approximately four-fold difference in the fluorescence ratios, is sufficient to distinguish two cell types (Fig. 1, bottom).

## Results

**Figure 1:**
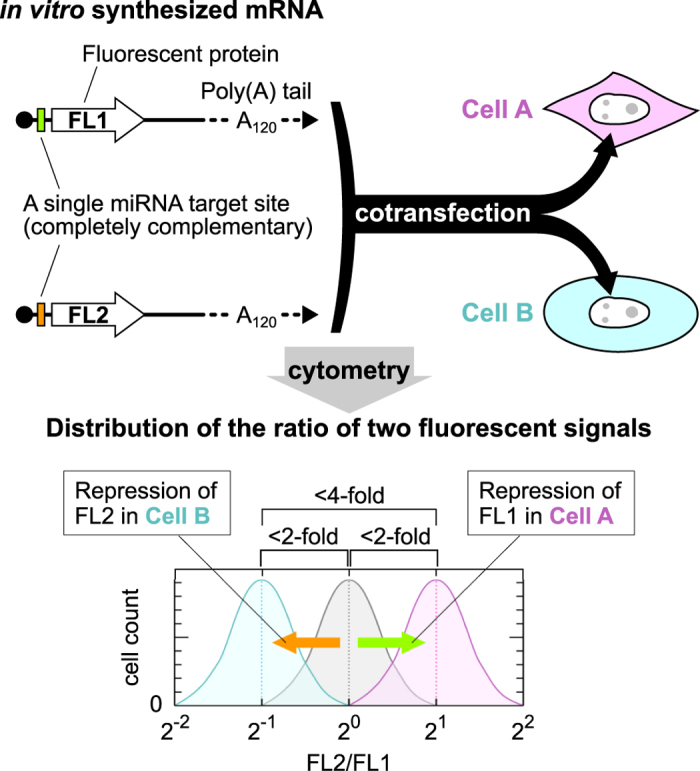
Schematic illustration of the high-resolution identification and separation of cell types. Synthesized a pair of mRNAs encoding fluorescent proteins (FL1 or FL2) contain distinct miRNA target site in the 5′ UTR (shown in green or orange), an anti-reverse cap analogue (black circle) at their 5′ end, and a poly (**A**) tail of 120 nucleotides in length (A120). Two mRNAs were mixed and cotransfected into two cell populations (Cell A and Cell B) via lipofection. Twenty-four hours after the transfection, the cells can be analysed with a flow cytometer or a cell image analyser. The bottom panel shows a schematic histogram of the FL2/FL1 fluorescence signal ratios in transfected cells A (magenta) and B (cyan). A grey peak denotes the position of the cells with control mRNAs that lack miRNA target sites. Cell A and Cell B are separated based on less than two-fold differences in miRNA activities.

**Figure 2:**
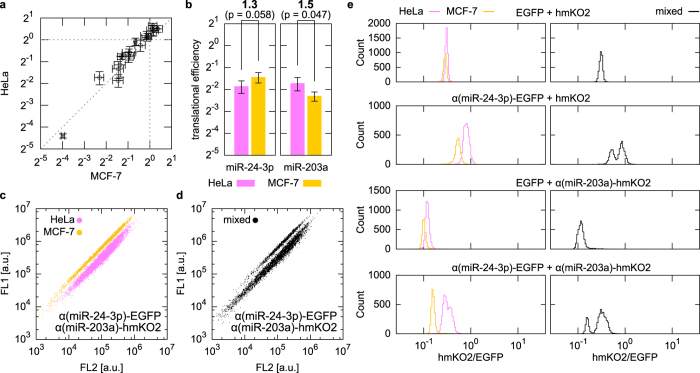
High-resolution identification and separation of HeLa cells and MCF-7 cells based on subtle differences in the activity of miR-24-3p (1.3-fold) and miR-203a (1.5-fold). (**a**) Comparison of translational efficiencies of 20 miRNA-responsive mRNAs between the two cell lines. Error bars indicate the mean ± standard deviation (n = 3) in each line. The translational efficiency of control EGFP mRNA, which does not contain a miRNA target site, was normalized to 1. See Supplementary Table S1 for details. (**b**) Translational efficiency of two selected miRNA-responsive mRNAs in HeLa (magenta) and MCF-7 (yellow) cells. The numbers on top indicate the fold-change of the efficiency between the two lines, followed by p-values within parentheses. Error bars indicate the mean ± standard deviation (n = 3). (**c**) Dot plots of individually transfected HeLa (magenta) and MCF-7 (yellow) cells. (**d**) Dot plot of a mixed HeLa and MCF-7 cell population that were transfected with the same pair of mRNAs shown in (**c**). (**e**) Histograms of the fluorescence ratio in HeLa cells (magenta), MCF-7 cells (yellow), or their mixture (black) transfected with indicated pairs of mRNAs. The fluorescence ratio was defined as FL2 (hmKO2) intensity divided by FL1 (EGFP) intensity for each cell.

To demonstrate our HRIC concept, we arbitrarily selected 20 miRNAs that have previously been examined in HeLa cells (human cervical cancer cells)^16^ and tested their activity in HeLa, MCF-7 (human breast cancer cells) and 293FT cells (human embryo kidney cells) using the 20 miRNA-responsive mRNAs that encode enhanced green fluorescent protein (EGFP) (Supplementary Table S1). We compared the miRNA activities between HeLa and MCF-7 cells, and found no miRNAs whose activity differed more than two-fold (Fig. 2a). Thus, we used HeLa and MCF-7 cells as a model case to distinguish cells with similar miRNA profiles. We investigated whether the cotransfection of two distinct miRNA-responsive mRNAs could separate HeLa and MCF-7 cells based on less than two-fold differences in the miRNA activities. We chose miR-24-3p and miR-203a, which were 1.3-fold more active and 1.5-fold less active in HeLa than MCF-7 cells, respectively (Fig. 2b). The cotransfection of these two miRNA-responsive mRNAs that encode either EGFP or Kusabira-Orange (hmKO2) (referred to as α(miR-24-3p)-EGFP and α(miR-203a)-hmKO2, respectively) distinguished the two cell types, whereas cotransfection of either of the mRNAs did not (Fig. 2c,e, left). In addition, the direct transfer of the two miRNA-responsive mRNAs into cocultured HeLa and MCF-7 cells clearly divided the mixed population into two groups (Fig. 2d,e, right). To investigate generality of our HRIC approach, we swapped the miRNA target sites of two mRNAs. The swapped pair (α(miR-203a)-EGFP and α(miR-24-3p)-hmKO2) also separated the two cell lines (Supplementary Fig. S2). Furthermore, we applied HRIC for separating 293FT cells from HeLa cells or MCF-7 cells to prove our concept. Similarly, transfection of appropriate miRNA-responsive mRNA pairs clearly distinguished HeLa and 293FT cells or MCF-7 and 293FT cells based on subtle differences (1.6- to 1.9-fold) in target miRNA activities between these cell types (Supplementary Fig. S3). These results support our idea that the combination of two miRNA-responsive mRNAs improves the resolution for cell separation by shifting the two peaks in opposite directions (Fig. 1). To our knowledge, we are the first to demonstrate that subtle difference of the miRNA activities (i.e., less than two-fold differences) between different cell types is sufficient to identify and separate target live cells. However, we could not distinguish the three cell types (HeLa, 293FT and MCF-7 cells) simultaneously by using any two mRNAs that respond to the tested miRNAs.

**Figure 3:**
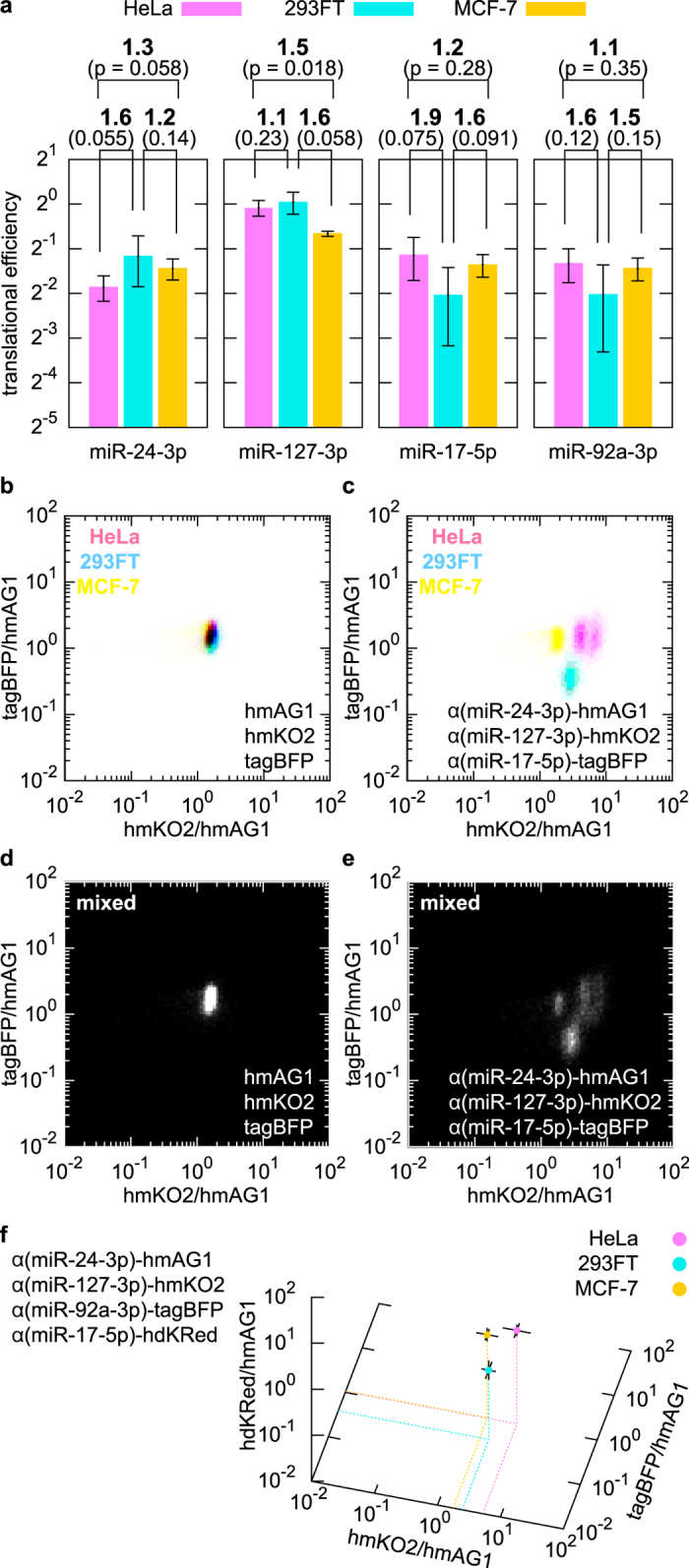
High-resolution identification and separation of three cell types using three miRNA-responsive mRNAs. (**a**) Translational efficiency of three miRNA-responsive mRNAs in HeLa (magenta), 293FT (cyan), and MCF-7 (yellow) cells, shown as in Fig. 2b. (**b**,**c**) 2-D densities of HeLa, 293FT and MCF-7 cells individually transfected with either control reporter mRNAs (**b**) or the three indicated miRNA-responsive mRNAs (**c**). The flow cytometry data are plotted against two fluorescence ratios: hmKO2 or tagBFP intensity divided by hmAG1 intensity. (**d**,**e**) Separation of mixed cells in 2-D space. The three lines were cocultured and transfected with the same mRNA set as in (**b**) or (**c**). (**f**) 3-D separation using four miRNA-responsive mRNAs. The geometric mean values of three ratios (intensities of hmKO2, tagBFP or hdKRed divided by the intensity of hmAG1 in each cell) were calculated and plotted. The distribution of the cells is shown in Supplementary Fig. S7.

Next, we considered that the transfection of more miRNA-responsive mRNAs into the same cell would improve the resolution of cell identification and separation (Supplementary Fig. S4). According to the translational efficiencies individually determined in HeLa, 293FT and MCF-7 cells (Supplementary Table S1), we evaluated whether sets of three miRNA-responsive mRNAs could be used to distinguish the three cell types simultaneously. From this analysis, we predicted that a combination of three mRNAs that individually respond to miR-24-3p, miR-127-3p and miR-17-5p, the activity of which differed by less than two-fold among cell types (Fig. 3a), could separate the three cell lines simultaneously. We synthesized the three miRNA-responsive reporter mRNAs, which coded distinct fluorescent proteins (Azami-Green (hmAG1), hmKO2 or tagBFP), and cotransfected them into individual cell lines. The fluorescence of hmKO2 or tagBFP in a transfected cell were divided by that of hmAG1 and plotted on a two-dimensional (2-D) plane as cell density (Fig. 3b–e). Upon transfection with these mRNAs, the three individual cell types formed specific, separate clusters (Fig. 3c), but not when transfected with control reporter mRNAs that lacked the miRNA target sequences (Fig. 3b). Transfection of the same set of miRNA-responsive mRNAs into a mixture of the three cell types also resulted in three separate clusters, the positions of which were consistent with those derived from the individual lines (Fig. 3e). Moreover, we found that HeLa cells could be divided into two subgroups by these mRNAs (Fig. 3c,e). Their distribution changed dependent on the culture condition (Supplementary Fig. S5), though profiles of short tandem repeats^17^ in their genome were identical (Supplementary Table S2 and Supplementary Fig. S6). These results suggest heterogeneity of the miRNA activity in HeLa cell populations and the level of resolution achievable by the HRIC method. The addition of one more reporter mRNA, which expressed another fluorescent protein, Keima-Red (hdKRed), resulted in one more fluorescence ratio and one more dimension for the separation space. Thus, using four miRNA-responsive mRNAs (Fig. 3a), the three cell lines (individual or cocultured cells) were clearly distinguishable in 3-D space based on three fluorescence ratios (Fig. 3f and Supplementary Fig. S7). To investigate possible cell separation space using multiple miRNA-responsive mRNAs, we transfected two-fold dilution series of control mRNAs that mimics repression by miRNA (Supplementary Table S3). The obtained density plot suggests that specific sets of three or four miRNA-responsive mRNAs have the potential to distinguish dozens or hundreds of cell types, respectively (Supplementary Fig. S8).

**Figure 4:**
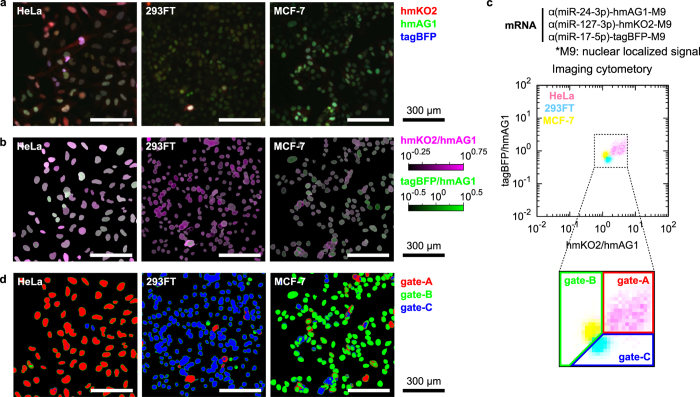
High-resolution identification of cell types with imaging cytometry. (**a**) Fluorescent images of HeLa, 293FT or MCF-7 cells transfected with α(miR-24-3p)-hmAG1-M9, α(miR-127-3p)-hmKO2-M9 and α(miR-17-5p)-tagBFP-M9. Signals from hmKO2, hmAG1 and tagBFP were merged into red, green and blue channels, respectively. Scale bars denote 300 μm. (**b**) Processed images with pseudo-colours of the two ratios in the nuclei: hmKO2/hmAG1 (purple) and tagBFP/hmAG1 (green). (**c**) 2-D density of the three transfected lines obtained by imaging cytometry. The magnified region inside the dotted box shows how we partitioned data to specify HeLa (gate-A), MCF-7 (gate-B) and 293FT (gate-C) cells shown in (**d**). (**d**) Cell classification of the images. According to the fluorescence ratios, pixels in the images were pseudo-coloured with red (gate-A), green (gate-B) or blue (gate-C).

Finally, we examined the versatility of our HRIC approach by using a cell image analyser that is able to maintain the spatial information of target cells, such as the shape and position. To identify each cell in an image, we fused reporter fluorescent proteins with a nuclear localized signal (M9). We observed differently coloured nuclei from the three cell lines transfected with a set of three miRNA-responsive mRNAs (Fig. 4a). The intensity of the nuclei varied among cells because of the variation in mRNA transfection efficiency. Therefore, we calculated the fluorescence ratio of each pixel in the nuclei in a manner similar to that of the flow cytometry analysis (Fig. 3b–e) and pseudo-coloured the ratios of hmKO2/hmAG1 and tagBFP/hmAG1 (Fig. 4b). Importantly, the averaged ratios of each nucleus in a density plot revealed that these three lines were clearly separated, consistent with flow cytometry analysis (compare Figs 3c with 4c). Gating the fluorescence ratios of the nuclei identified the three living cell types in the images simultaneously (Fig. 4d). These results indicate that our HRIC method can be applied to not only flow cytometry but also imaging cytometry.

## Discussion

We developed a method to identify and separate live mammalian cells by quantifying multiple miRNA activities between heterogeneous cell populations. Our HRIC approach, which distinguishes cells with less than two-fold difference in miRNA activity, improves the resolution for identifying different cell types. We assume that such precision in measuring miRNA activity is caused by the nature of the protein production from the *in vitro* synthesized mRNAs transfected into the cells. In the case of canonical DNA induction methods, a smaller number of DNA molecules in the nucleus should express a sufficient level of reporter proteins through both transcription and translation processes. In contrast, in the case of mRNA transfection, a larger number of mRNA molecules are likely to be transferred into the cytoplasm and produce proteins via only translation. As a result, the fluorescence ratio between two reporter proteins translated from two synthetic mRNAs cotransfected into the same cell is almost constant among individual cells (Supplementary Fig. S1), and shows little stochastic noise irrespective of the varying fluorescence levels between cells.

The variation in fluorescence among transfected cells mainly results from the amount of synthetic mRNAs taken into each cell. Detection of miRNA activity by the mRNAs shifts in parallel the broadly distributed cell populations on dot plots (Compare Fig. 2c and S2c with S1a), which maintains sharp peaks of the populations in histogram of the fluorescence ratio. These observations indicate that the efficiency of repression by the miRNAs is constant among the transfected cells irrespective of their mRNA uptake and suggest turnover of miRNA functions. Our synthetic mRNAs that contain completely complementary sequences for the target miRNAs could be cleaved by endogenous miRNAs with Argonaute2^18^.

In addition to detecting intracellular information, the transient nature of the synthetic mRNAs avoids potential risks of genomic integration and resulting genetic defects caused by a plasmid or a viral vector^19^ and is unlikely to affect the activities of endogenous miRNAs^15^, which enables us to purify specific cells for downstream applications, such as cell line establishment, transcriptome profiling analysis, *in vitro* cell differentiation and cell therapeutics. In this study, each of the reporter mRNAs was designed to respond to one target miRNA, and thus, at most, four intracellular parameters were detected simultaneously (Fig. 3f). Expanding the method with multiple miRNA inputs/responsive mRNAs that include different target miRNA sequences^15^,^20^,^21^ and with a gene circuit that performs required mathematical operations^22^ may further improve the resolution of cell identification based on higher-order intracellular information.

## Methods

### Oligonucleotides

All primer and oligo-DNA sequences used in this study are listed in Supplementary Tables S1 and S4.

### Plasmid construction

An *in vitro* transcription (IVT) template for tagRFP (Evrogen, Moscow, Russia) mRNA was amplified via PCR using the primer set T7Fwd5UTR and Rev120A from the plasmid pSRT-tagRFP. First, pGEM T-easy (Promega, Madison, WI, USA) was modified via PCR-based site-directed mutagenesis with the primer set FwdMCS and RevMCS to obtain a pAM empty vector. Next, a DraI-digensted fragment of pAM was ligated with the blunted NheI and AgeI sites of pCDFDuet-1 (Novagen, Billerica, MA, USA) to create the pSM empty vector. To create the pSRT empty vector, a pair of oligo-DNAs, Code5UTR and Comp5UTR, were annealed and inserted into the EcoRI-NcoI site, followed by the insertion of annealed Code3UTR and Comp3UTR into the XbaI-HindIII site. Finally, the coding sequence of tagRFP or tagBFP (Evrogen) was amplified via PCR with the appropriate set of primers (CFwdtagRFP and CRevtagRFP, or CFwdtagBFP and CRevtagBFP), digested with NcoI and BglII and inserted into the NcoI-BglII site of pSRT to generate pSRT-tagRFP or pSRT-tagBFP, respectively.

To fuse fluorescent proteins with nuclear localized signals, M9, the coding regions of hmAG1 (Amalgaam, Tokyo, Japan) and hmKO2 (Amalgaam) were amplified with the appropriate primer sets (CFwdhmAG1 and CRevhmAG1, and CFwdhmKO2 and CRevhmKO2) to replace the region of tagBFP in pSRT-tagBFP. Then, the M9 sequence was amplified via PCR with FwdM9 and RevSV40 from p4LambdaN22-3mEGFP-M9^23^, digested with BamHI and BglII and inserted into the BglII sites of the resulting vectors as well as pSRT-tagBFP.

As summarized in Supplementary Table S1, some 5′ UTR fragments were generated from plasmids (“plasmid” in “Construction”). Pairs of the indicated DNA fragments were annealed and inserted into the BamHI–AgeI site of 18nt-2xFr15-ECFP^24^. The 5′ UTR was amplified using the primer set T7FwdA and Rev5UTR.

### Preparation of template DNA for IVT

The protein-coding region of fluorescent proteins and CBG68luc (Promega) were amplified by PCR with the appropriate primer sets and plasmids (“ORF” in “Type of target”, Supplementary Table S4). The 5′ and 3′ UTR sequences not containing a miRNA target site (for control mRNAs) were amplified by PCR with the appropriate primer sets and synthesized oligo-DNAs (“5′ UTR” and “3′ UTR” in “Type of target”, Supplementary Table S4). Some miRNA-responsive 5′ UTRs were prepared via PCR amplification from the plasmids as described earlier (“plasmid” in “Construction”, Supplementary Table S1), and the others were derived from synthesized oligo-DNAs (“oligoDNA” in “Construction”, Supplementary Table S1). To generate a template for IVT, 50 ng of the reporter protein-coding DNA region and 10 pmol of each of the 5′ and the 3′ fragments were concatenated and amplified via PCR with the primer set T7FwdA and Rev120A.

At the end of the reaction, samples containing plasmid DNA were digested with DpnI restriction enzyme (Toyobo, Osaka, Japan) for 30 min at 37 °C. After purification with a MinElute PCR purification kit (Qiagen, Hilden, Germany), the PCR products were subjected to IVT.

### Synthesis and purification of mRNAs

All mRNAs were synthesized using a MegaScript T7 kit (Ambion, Carlsbad, CA, USA) with a modified protocol. In brief, uridine triphosphate and cytosine triphosphate were replaced with pseudouridine-5′-triphosphate and 5-methylcytidine-5′-triphosphate (TriLink BioTechnologies, San Diego, CA), respectively. As for guanosine triphosphate, a premix of guanosine triphosphate and Anti Reverse Cap Analog (New England Biolabs, Ipswich, MA) (1:4) was used. The reaction mixtures were incubated at 37 °C for 4 h and further incubated at 37 °C for 30 min in the presence of TURBO DNase (Ambion). RNA products were purified with a FavorPrep Blood/Cultured Cells total RNA extraction column (Favorgen Biotech, Ping-Tung, Taiwan) according to the manufacture’s protocol and then subjected to treatment with Antarctic Phosphatase (New England Biolabs) at 37 °C for 30 min. Finally, the resulting mRNAs were purified again using an RNeasy MiniElute Cleanup Kit (Qiagen).

### Cell culture

HeLa (originally obtained from ATCC) and MCF-7 cells were cultured in Dulbecco’s modified Eagle’s Medium (DMEM)-F12 and RPMI 1640 medium, respectively, containing 10% fetal bovine serum (FBS) and 1% antibiotic-antimycotic solution (Sigma-Aldrich, St. Louis, MO, USA). 293FT cells (Invitrogen, Carlsbad) were grown in DMEM supplemented with 10% FBS, 2 mM L-glutamine (Invitrogen), 0.1 mM non-essential amino acids (Invitrogen), 1 mM sodium pyruvate (Sigma-Aldrich) and 0.5% penicillin-streptomycin (Invitrogen). In the cell-mixing assays, each cell was prepared in the same medium as HeLa cells, and equal numbers of cells were mixed and seeded.

### Transfection of mRNAs

Transfection of mRNAs was performed on 24-well plates using 1 μL of StemFect (Stemgent, Cambridge, MA) according to the manufacturer’s instructions. The day before the transfection, cultured cells were seeded onto 24-well plates. The medium was changed 4 h after the transfection. To measure the translational efficiency, 100 ng each of reporter EGFP mRNA responsive to a miRNA and control tagRFP mRNA were transfected. To separate cells using two miRNA-responsive mRNAs, 100 ng and 125 ng of reporter mRNAs expressing EGFP or hmKO2, respectively, were transferred into target cells or their mixture. In dilution assay (Fig. S8), CBG68luc mRNA was used to dilute hmAG1, hmKO2 and tagBFP mRNAs. The transfection conditions of each experiment are summarized in Supplementary Table S5.

### Flow cytometry

Prior to flow cytometry, transfected cells were detached from a dish and filtered through a nylon mesh. An Accuri C6 flow cytometer (BD Biosciences, San Jose, CA) with FL1 (530/30 nm) and FL2 (585/40 nm) filters was used in the analysis of cotransfection of 2 mRNAs: EGFP and hmAG1 were detected with FL1, and tagRFP and hmKO2 with FL2. FACSAria II (BD Biosciences) was used in experiments involving 3 or 4 mRNAs: hmAG1, hmKO2, tagBFP and hdKRed were detected with a blue laser (488 nm) with an FITC filter (530/30 nm), a green laser (561 nm) with a PE filter (585/42 nm), a violet laser (405 nm) with a Pacific Blue filter (450/40 nm) and a violet laser with a Qdot 605 filter (610/20 nm), respectively. Dead cells and debris were excluded by forward and side scatter signals. Coloured density plots (Figs 3b,c and 4c and S7) were generated as negative pictures of merged density plots in which the densities of 293FT, HeLa, and MCF-7 cells are shown in the red, green, and blue channels, respectively.

### Measurement of translational efficiency

Cells were transfected with 100 ng of miRNA-responsive EGFP mRNA with 100 ng of tagRFP mRNA. Twenty-four hours after the transfection, the transfected cells were subjected to flow cytometry using the Accuri C6 flow cytometer. The fluorescence intensity of EGFP and tagRFP were compensated based on a spectral matrix^25^ generated from data sets of cells transfected with either EGFP or tagRFP^26^. The mean fluorescence ratio was obtained as the mean intensity of compensated EGFP signals divided by that of compensated tagRFP signals. The translational efficiency was defined as the mean fluorescence ratio of miRNA-responsive EGFP mRNA normalized by that of EGFP mRNA that does not contain a miRNA target site. P-values indicate the probability of t-tests for unequal variance.

### Prediction of a set of miRNA-responsive mRNAs

According to the translational efficiency of 20 miRNA-responsive mRNAs in three cell lines (HeLa, MCF-7, and 293FT) (Supplementary Table S1), the distance between cell types *A* and *B* (*d*_*A,B*_) made by a set of three miRNA-responsive mRNAs, α(*x*)-hmAG1, α(*y*)-hmKO2 and α(*z*)-tagBFP, was predicted as follows:


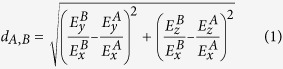


where 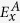 indicates the translational efficiency of α(*x*)- hmAG1 measured in cell type *A*. Sets of three miRNA-responsive mRNAs were scored by the minimum distance among HeLa, 293FT and MCF-7 cells, and high-scored mRNA sets were subjected to transfection experiments. The prediction for four mRNAs (the three aforementioned mRNAs plus α(*w*)-hdKRed) was performed in a similar manner by using the following equation:


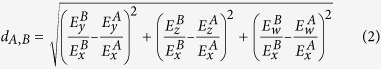


### Imaging cytometry

Reporter fluorescent proteins were fused with nuclear localized signals (M9) to identify transfected cells. Twenty-four hours after transfecting three mRNAs, brightfield images and fluorescent images of the cells were captured using an IN Cell Analyzer 6000 (GE Healthcare Bio-sciences, Piscataway, NJ). The fluorescence signals of hmAG1, hmKO2 and tagBFP were detected using a blue laser (488 nm) with an FITC filter (525/36 nm), a green laser (561 nm) with a DsRed filter (605/52 nm) and a UV laser (405 nm) with a DAPI filter (455/50 nm), respectively. The obtained images were analysed using CellProfiler software^27^. First, nuclei were identified in the average image of three fluorescence channels. Next, similar to flow cytometry, the hmKO2 signal and the tagBFP signal in each pixel were divided by the hmAG1 signal. Then, the geometric mean of the ratios was calculated in each nucleus. In making pseudo-coloured images, the range of the ratios (hmKO2/hmAG1, 10^−0.25^ to 10^0.75^; tagBFP/hmAG1, 10^−0.5^ to 10^0.5^) was normalized to a 0 to 1 scale prior to merging the images in purple (red and blue) and green channels. Images were edited using ImageJ software (NIH, Bethesda, MD, USA).

### Short tandem repeat profiling

Genomic DNAs were extracted from freeze stocks of HeLa cells (1–3 × 10^6^ cells), cultured 293FT and MCF-7 cells (5 × 10^6^ cells) with GenElute Mammalian Genomic DNA Miniprep Kit (Sigma-Aldrich) according to manufacturer’s instructions. From the genomic DNAs (10 ng), 10 genomic loci were amplified using GenePrint 10 System (Promega) and analysed by 3130 Genetic Analyzer (Applied Biosystems, Carlsbad, CA, USA).

## Additional Information

**How to cite this article**: Endo, K. *et al*. High-resolution Identification and Separation of Living Cell Types by Multiple microRNA-responsive Synthetic mRNAs. *Sci. Rep.* **6**, 21991; doi: 10.1038/srep21991 (2016).

## Supplementary Material

Supplementary Information
