# Supplementary material for: High-resolution Identification and Separation of Living Cell Types by Multiple microRNA-responsive Synthetic mRNAs
Source: Sci Rep. 2016 Feb 23;6:21991. doi: 10.1038/srep21991 (PMC4763229; doi:10.1038/srep21991)
Supplement: Supplementary Information [file srep21991-s1.pdf]

Supplementary information

High-resolution Identification and Separation of Living Cell Types by Multiple microRNA-responsive Synthetic mRNAs

Kei Endo*, Karin Hayashi, Hirohide Saito*

Contents:

1. **Supplementary Figures 1–8**
2. **Supplementary Tables 1–5**

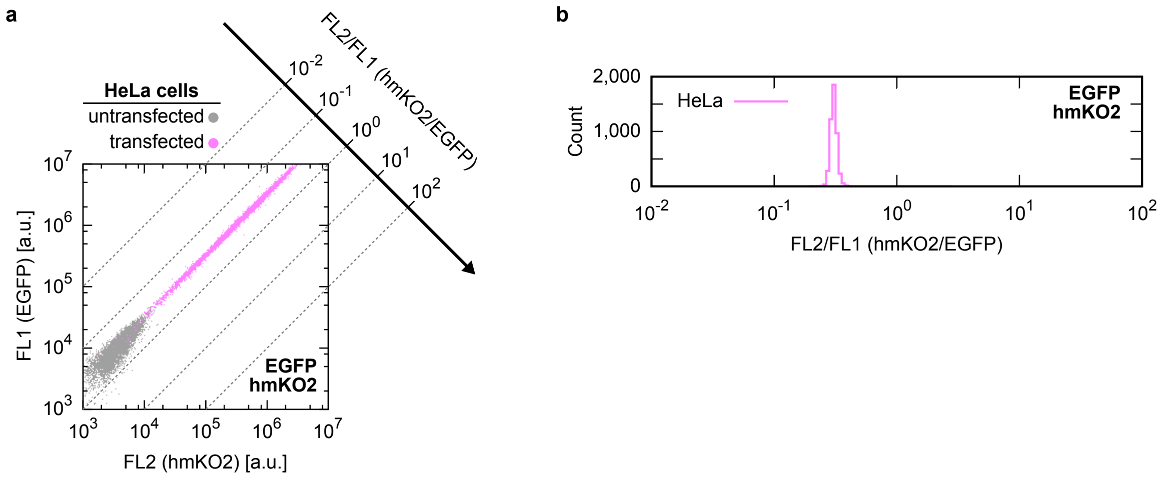

Supplementary Figure S1. Detecting a cell population as a peak in a histogram of fluorescence ratio. (a) Dot plot of HeLa cells that were transfected with (transfected, magenta) or without (untransfected, gray) EGFP and hmKO2 mRNAs. The cells were analysed with a flow cytometer 24 h after the transfection. Due to high efficiency of mRNA transfection, almost all the transfected cells in a dish showed both fluorescence signals. Dotted lines denote scales for the fluorescence ratio, FL2/FL1. (b) A fluorescence ratio histogram of the transfected HeLa cells shown in a. The cells can be detected as a sharp peak.

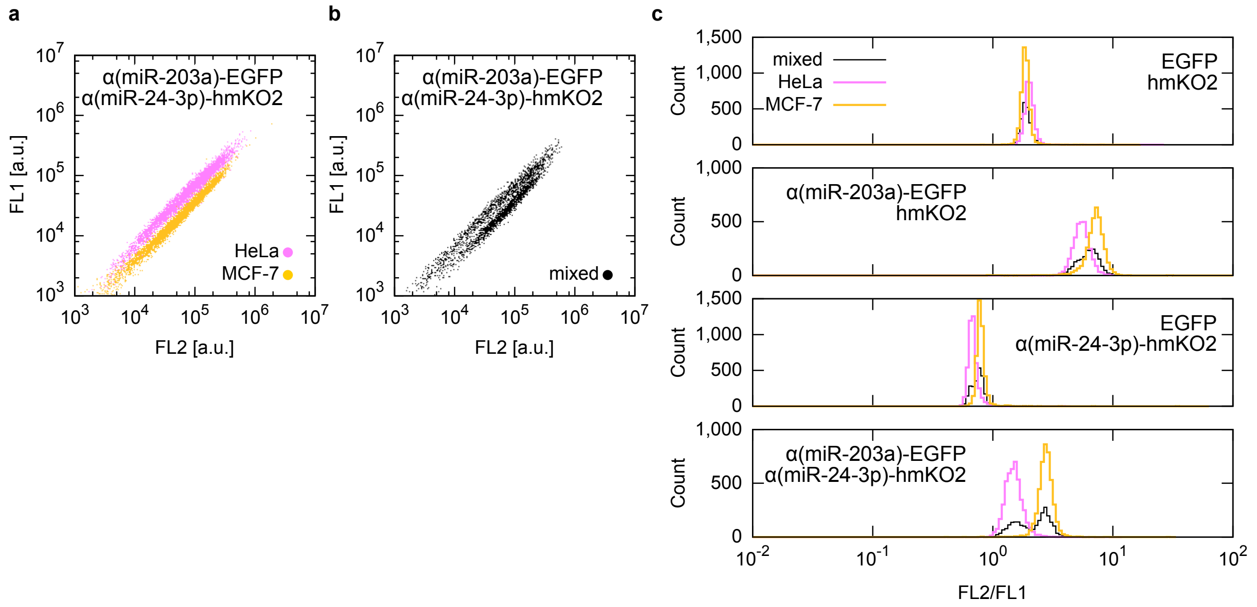

Supplementary Figure S2. Separation of HeLa and MCF-7 cells with a swapped pair of miRNA-responsive mRNAs. (a) A dot plot of HeLa (magenta) and MCF-7 (yellow) cells that were individually transfected with an indicated pair of miRNA-responsive mRNAs. The cells were analysed by flow cytometry 24 h after the transfection. (b) A dot plot of the mixture of HeLa and MCF-7 cells transfected with the pair of mRNAs. The cells were analysed by flow cytometry 24 h after the transfection. (c) Histograms of the fluorescence ratio. Individual HeLa (magenta) and MCF-7 (yellow) cells and their mixtures (black) were transfected with the indicated pairs of mRNAs. The ratio of two fluorescence signals (FL2/FL1) in each cell as measured by flow cytometry was calculated and plotted. The swapped mRNA pair separated HeLa cells and MCF-7 cells, but less efficiently than the original pair shown in Figure 2. The results indicate that the combination of miRNA target sites and reporter fluorescent proteins has some impact on the efficiency of HRIC to distinguish cells.

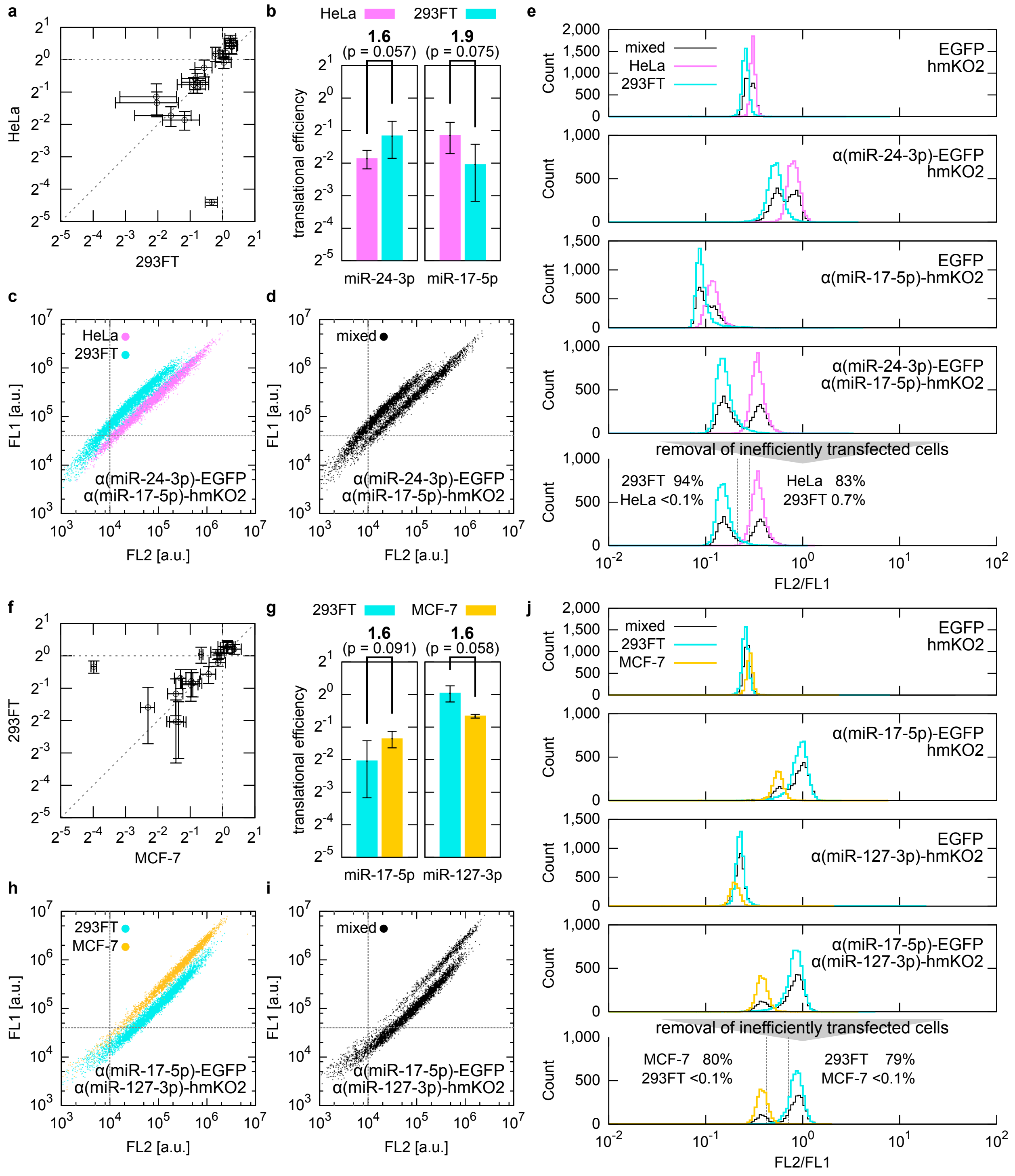

Supplementary Figure S3. High-resolution identification and separation of two cell lines using two miRNA-responsive mRNAs. Specific pairs of miRNA-responsive mRNAs, the activity of which differed by less than two-fold, were capable of distinguishing 293FT (cyan)

from HeLa (magenta) (**a**–**e**) and MCF-7 (yellow) (**f**–**j**) cells. (a, f) Comparison of translational efficiencies between the two cell lines. Error bars indicate the mean ± standard deviation (n=3) in each line. The translational efficiency of the control EGFP mRNA, which does not contain a miRNA target site, was normalized to 1. The data set is provided in Table S1. (b, g) Translational efficiency of two mRNAs responsive to the indicated miRNAs in each line. The numbers on top are the fold-change between the cell lines, followed by p-values within parentheses. Error bars indicate the mean ± standard deviation (n=3). (c, d, h, i) Dot plots of transfected cells. The indicated cell lines (**c**, **h**) and their mixed population in a dish (black; **d**, **i**) were transfected with the indicated miRNA-responsive mRNAs and analysed by flow cytometry 24 h after the transfection. Dotted lines denote criteria for removal of inefficiently transfected cells (FL1 > 40,000 and FL2 > 10,000). (e, j) Histograms of the fluorescence ratios. Individual cell lines and their mixtures (black) were transfected with the indicated pairs of mRNAs. The ratio of two fluorescence signals (FL2/FL1) in each cell as measured by flow cytometry was calculated and plotted. The most bottom panels show possible criteria for purification of each cell line with the two miRNA-responsive mRNAs. The criteria are shown as dotted line, and labelled with yields of expected and unexpected cells. In these panels, the inefficiently transfected cells are removed to improve the purity of cells of interest. We can improve resolution of cell separation by using two miRNA-responsive mRNAs.

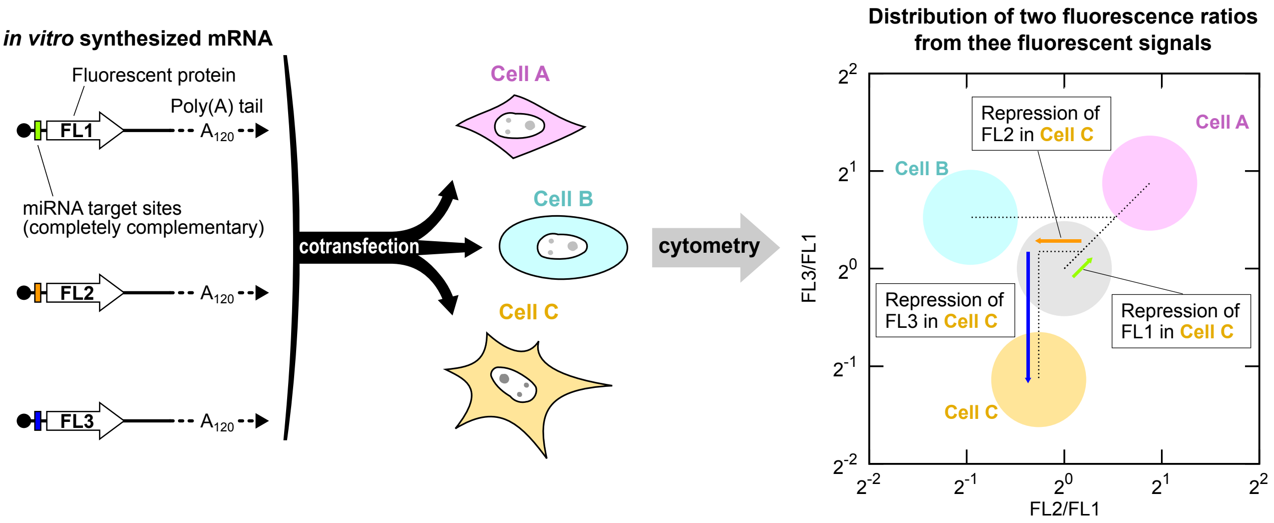

Supplementary Figure S4. Schematic illustration of two-dimensional (2-D) separation using three miRNA-responsive mRNAs. Three reporter mRNAs expressing a different fluorescent protein (FL1, FL2 or FL3) and each containing one complementary sequence of one of three miRNAs (miRNA target site) in their 5' UTR were synthesized *in vitro*. Synthetic mRNAs contain an anti-reverse cap analogue (black circle) and a poly(A) tail (120 nucleotides). The mRNAs were mixed and co-transferred into target cells (Cell A, B and C) via lipofection. Twenty-four hours later, the cells were analysed with a flow cytometer. The right panel shows a schematic density plot of the cells using two fluorescence signal ratios (FL2/FL1 and FL3/FL1). Cells A, B and C are shown in magenta, cyan and yellow, respectively. A gray spot denotes the position of the cells with control mRNAs that were not affected by the miRNAs. As an example, the arrows in the plot indicate movement of cell C in accordance with the repression of the three reporter mRNAs by the three different miRNAs.

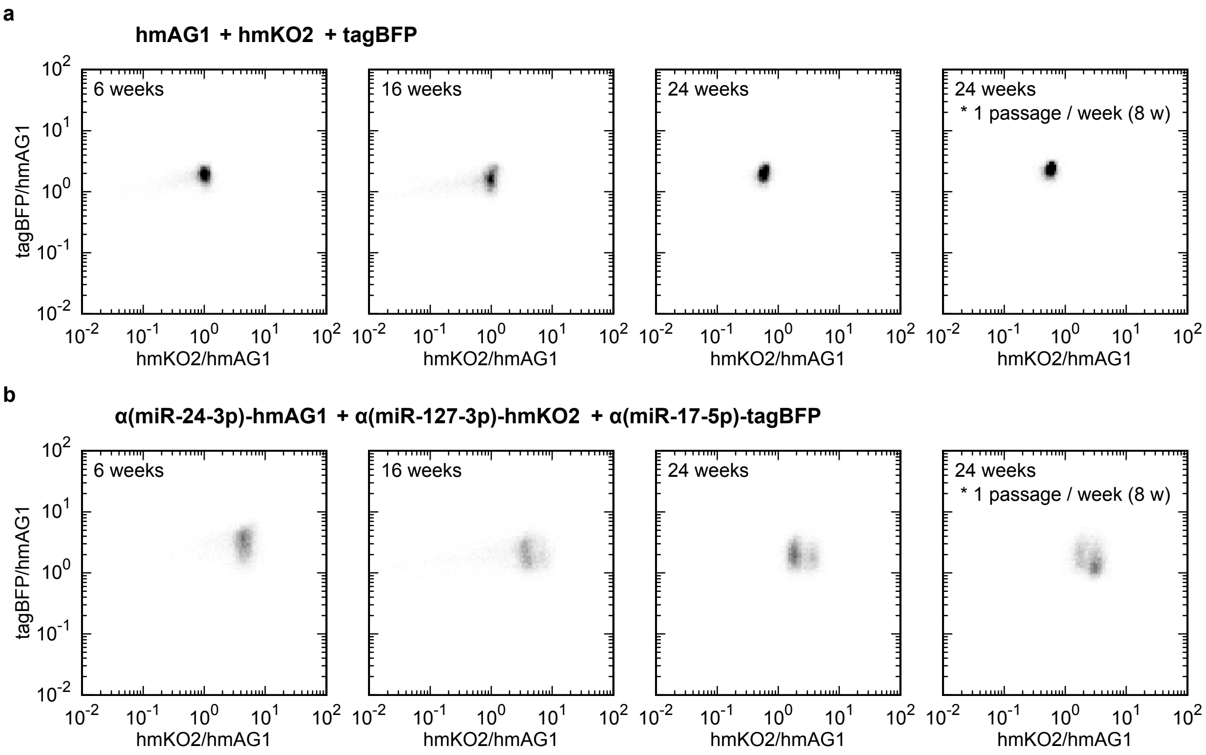

Supplementary Figure S5. Distribution of subgroups in HeLa cells. HeLa cells were maintained for 6, 16 and 24 weeks with three passages a week, and then transfected with the control set of three reporter mRNAs (hmAG1, hmKO2 and tagBFP) (**a**) or a specific set (**b**) of three miRNA-responsive reporter mRNAs. In the most right panels, HeLa cells were maintained 16 weeks with three passages a week and then 8 weeks with one passage a week (total 24 weeks). The corresponding mRNAs were transfected into cells at the indicated time, and the transfected cells were analysed with a flow cytometer 24 h after the transfection. Results are shown as a 2-D density plot of two fluorescence ratios. Please note that cell distribution balance shown in **b** was dependent on the number of cell passages and the culture condition (24 weeks).

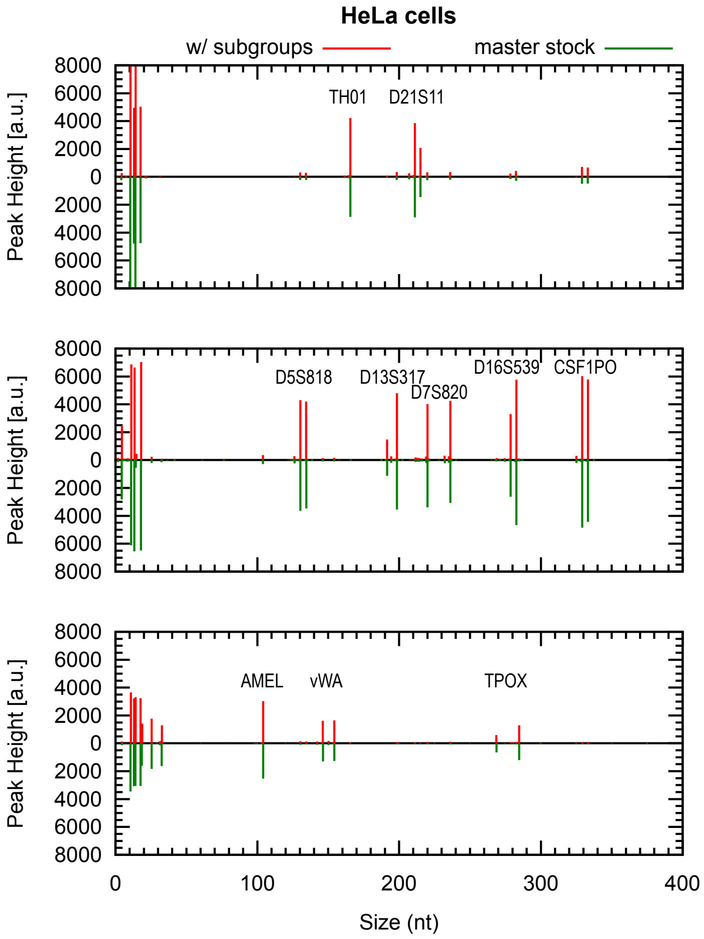

Supplementary Figure S6. Comparison of HeLa cell populations in STR profiling. HeLa cells composed of subgroups ("w/ subgroups", red, upward) and our master stock of HeLa cells that was made after five passages from purchased HeLa cells ("master stock", green, downward) were subjected to STR profiling of 10 genomic loci. Height and estimated size of detected peaks of amplified fragments are plotted. STR profiles of these two samples are identical to one another.

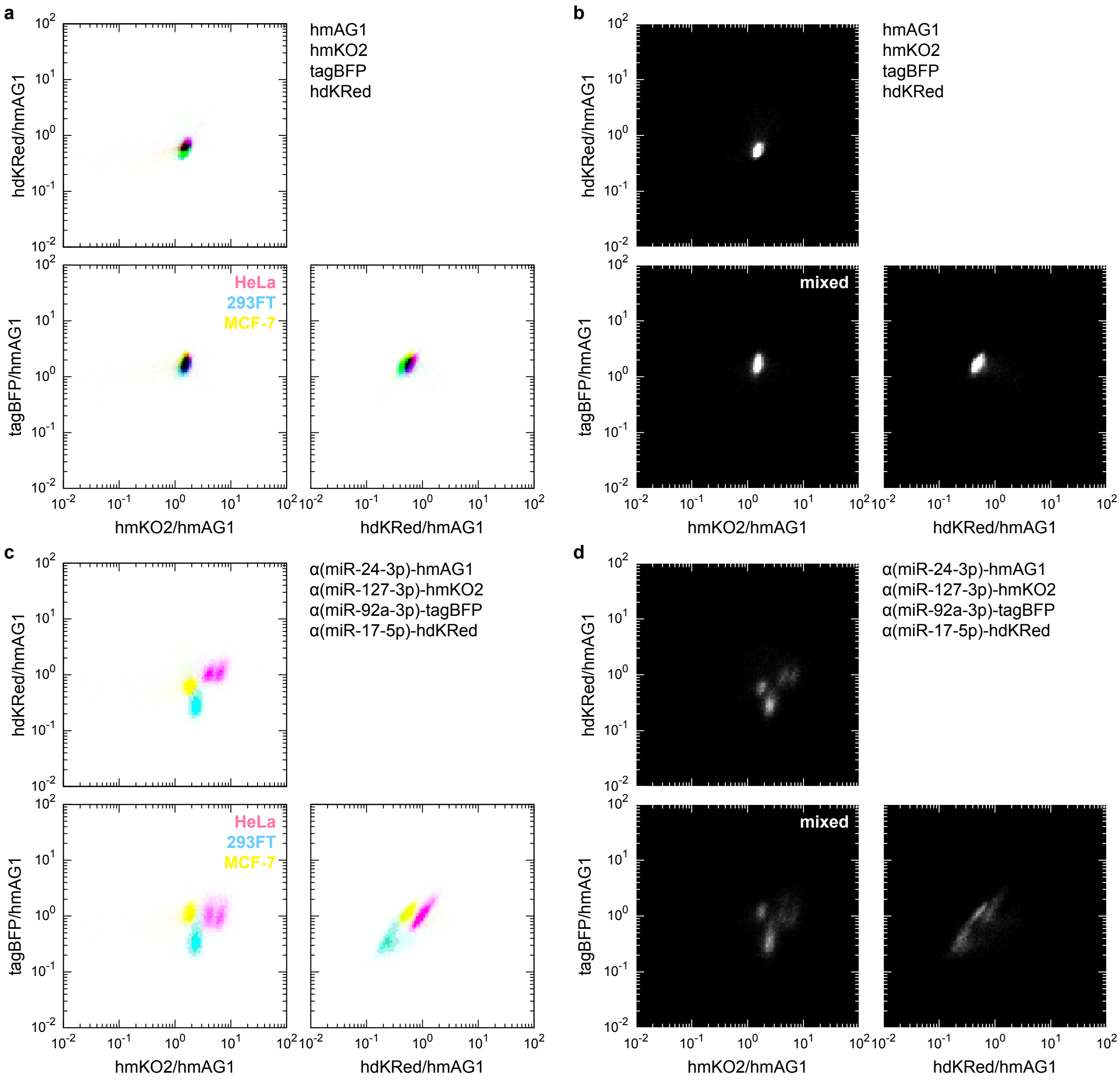

Supplementary Figure S7. 3-D separation using 4 miRNA-responsive mRNAs. Sets of 2-D density plots for 3-D separation. HeLa cells (magenta), 293FT cells (cyan), MCF-7 cells (yellow) (**a**, **c**) or their mixture (**b**, **d**) were transfected with the control (**a**, **b**) or a set of four miRNA-responsive reporter mRNAs (**c**, **d**). The cells were analysed by flow cytometry at 24 h after the transfection. Densities of the cells are plotted on the three planes vertical to the three axes using the fluorescence ratios. Representative results from three independent experiments are shown.

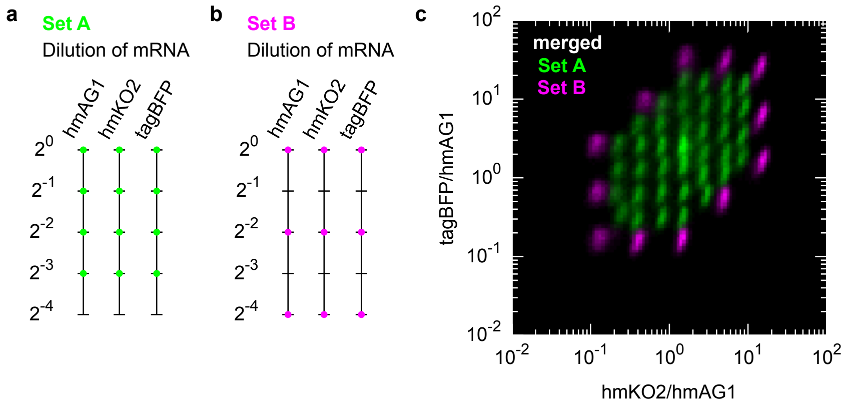

Supplementary Figure S8. 2-D separation space using 3 fluorescent reporter mRNAs. Control hmAG1, hmKO2 and tagBFP mRNAs were serially diluted in two-fold steps and transfected into HeLa cells in various combinations. The dilution of mRNAs mimics the repression by miRNAs in a cell. (a) Combination of the three mRNAs in Set A. Set A covers 2-fold dilution in 4 steps (2^0, 2^{-1}, 2^{-2}, 2^{-3}). A combination of the three mRNAs in 2-fold dilution series were tested (See Supplementary Table S3). (b) Combination of the three mRNAs in Set B. Set B includes the 1st, 3rd, and 5th steps of the 2-fold dilution (2^0, 2^{-2}, 2^{-4}). (c) A 2-D density plot of two fluorescence ratios. HeLa cells were transfected with various combinations of the mRNAs, and 24 h later analysed by a flow cytometer. The averaged density plot of the results from 38 (green, Set A) and 12 (purple, Set B) transfections are merged. Detailed conditions for the transfections are provided in Supplementary Table S3.

Supplementary Table S1. List of miRNA-responsive mRNAs used in this study and their translational efficiencies and primers for construction.

miRNA	HeLa	293FT	MCF-7	Construction	Primer (Pair)	
Name (miRBase ID)	Translational efficiency				Name(s)	Sequence(s)
hsa-miR-133a (MIMAT0000427)	0.842	0.677	0.743	plasmid	5Tmi133a_Afwd / 5Tmi133a_Arev	GATCCCAGCTGGTTGAAGGGGACCAAAAGATCTA / CCGGTAGATCTTTTGGTCCCCTTCAACCAGCTGG
hsa-miR-17-3p (MIMAT0000071)	1.131	1.002	0.928	plasmid	5Tmi17_3_Afwd / 5Tmi17_3_Arev	GATCCCTACAAGTGCCTTCACTGCAGTAGATCTA / CCGGTAGATCTACTGCAGTGAAGGCACTTGTAGG
hsa-miR-17-5p (MIMAT0000070)	0.451	0.242	0.389	plasmid	5Tmi17_5_Afwd / 5Tmi17_5_Arev	GATCCCTACCTGCACTGTAAGCACTTTGAGATCTA / CCGGTAGATCTCAAAGTGCTTACAGTGCAGGTAGG
hsa-miR-1 (MIMAT0000416)	1.675	1.328	1.345	plasmid	5Tmi1_Afwd / 5Tmi1_Arev	GATCCATACATACTTCTTTACATTCCAAGATCTA / CCGGTAGATCTTGGAATGTAAAGAAGTATGTATG
hsa-miR-206 (MIMAT0000462)	1.565	1.190	1.104	plasmid	5Tmi206_Afwd / 5Tmi206_Arev	GATCCCCACACACTTCCTTACATTCCAAGATCTA / CCGGTAGATCTTGGAATGTAAGGAAGTGTGTGGG
hsa-miR-21-5p (MIMAT0000076)	0.047	0.793	0.063	plasmid	5Tmi21_Afwd / 5Tmi21_Arev	GATCCTCAACATCAGTCTGATAAGCTAAGATCTA / CCGGTAGATCTTAGCTTATCAGACTGATGTTGAG
hsa-miR-367-3p (MIMAT0000719)	1.419	1.156	1.286	plasmid	5Tmi367_Afwd / 5Tmi367_Arev	GATCCTCACCATTGCTAAAGTGCAATTAGATCTA / CCGGTAGATCTAATTGCACTTTAGCAATGGTGAG
hsa-miR-373-5p (MIMAT0000725)	1.292	1.149	1.031	plasmid	5Tmi373_5_Afwd / 5Tmi373_5_Arev	GATCCGGAAAGCGCCCCCATTTTGAGTAGATCTA / CCGGTAGATCTACTCAAAATGGGGGCGCTTTCCG
hsa-miR-92a-3p (MIMAT0000092)	0.398	0.245	0.368	oligoDNA	5UTR-T92a-3p	CGACTCACTATAGGTCAGATCCGCTAGGATCCACAGGCCGGGA CAAGTGCAATAAGATCTACCGGTCGCCACCATG
hsa-miR-16-5p (MIMAT0000069)	0.587	0.620	0.406	oligoDNA	5UTR-T16-5p	CGACTCACTATAGGTCAGATCCGCTAGGATCCCGCCAATATTT ACGTGCTGCTAAGATCTACCGGTCGCCACCATG
hsa-miR-197-3p (MIMAT0000227)	0.667	0.557	0.528	oligoDNA	5UTR-T197-3p	CGACTCACTATAGGTCAGATCCGCTAGGATCCGCTGGGTGGAG AAGGTGGTGAAAGATCTACCGGTCGCCACCATG
hsa-miR-24-3p (MIMAT0000080)	0.275	0.444	0.368	oligoDNA	5UTR-T24-3p	CGACTCACTATAGGTCAGATCCGCTAGGATCCCTGTTCCTGCT GAACTGAGCCAAGATCTACCGGTCGCCACCATG
hsa-miR-339-5p (MIMAT0000764)	1.134	0.863	0.907	oligoDNA	5UTR-T339-5p	CGACTCACTATAGGTCAGATCCGCTAGGATCCCGTGAGCTCCT GGAGGACAGGGAAGATCTACCGGTCGCCACCATG
hsa-miR-224-5p (MIMAT0000281)	1.321	1.211	1.120	oligoDNA	5UTR-T224-5p	CGACTCACTATAGGTCAGATCCGCTAGGATCCAACGGAACCAC TAGTGACTTGAGATCTACCGGTCGCCACCATG
hsa-miR-127-3p (MIMAT0000446)	0.943	1.029	0.631	oligoDNA	5UTR-T127-3p	CGACTCACTATAGGTCAGATCCGCTAGGATCCAGCCAAGCTCA GACGGATCCGAAGATCTACCGGTCGCCACCATG
hsa-miR-365a-3p (MIMAT0000710)	1.074	1.082	0.907	oligoDNA	5UTR-T365a-3p	CGACTCACTATAGGTCAGATCCGCTAGGATCCATAAGGATTTT TAGGGGCATTAAGATCTACCGGTCGCCACCATG
hsa-miR-183-5p (MIMAT0000261)	1.355	1.222	1.108	oligoDNA	5UTR-T183-5p	CGACTCACTATAGGTCAGATCCGCTAGGATCCAGTGAATTCTA CCAGTGCCATAAGATCTACCGGTCGCCACCATG

hsa-miR-331-3p (MIMAT0000760)	0.584	0.539	0.508	oligoDNA	5UTR-T331-3p	CGACTCACTATAGGTCAGATCCGCTAGGATCCTTCTAGGATAG GCCCAGGGGCAGATCTACCGGTCGCCACCATG
hsa-miR-203a (MIMAT0000264)	0.302	0.331	0.202	oligoDNA	5UTR-T203a	CGACTCACTATAGGTCAGATCCGCTAGGATCCCTAGTGGTCCT AAACATTTCACAGATCTACCGGTCGCCACCATG
hsa-miR-214-3p (MIMAT0000271)	0.617	0.581	0.512	oligoDNA	5UTR-T214-3p	CGACTCACTATAGGTCAGATCCGCTAGGATCCACTGCCTGTCT GTGCCTGCTGTAGATCTACCGGTCGCCACCATG

Supplementary Table S2. STR profiling of the cell lines used in this study.

Locus	HaLa				293FT		MCF-7	
	w/ subgroups		Master stock					
TH01	7		7		7	9.3	6	
D21S11	27	28	27	28	30.2		30	
D5S818	11	12	11	12	8	9	11	12
D13S317	12	13.3	12	13.3	12	14	11	
D7S820	8	12	8	12	11		8	9
D16S539	9	10	9	10	9	13	11	12
CSF1PO	9	10	9	10	7	12	10	
AMEL	X		X		X		X	
vWA	16	18	16	18	16	19	14	15
TPOX	8	12	8	12	11		9	12

Supplementary Table S3. Combinations of diluted reporter mRNAs used in Figure S6.

Set	Dilution factor		
	hmAG1 (40 ng)	hmKO2 (10 ng)	tagBFP (200 ng)
**A**	1	1	1
**A**	1	1	2
**A**	1	1	4
**A**	1	1	8
**A**	1	2	1
**A**	1	2	2
**A**	1	2	4
**A**	1	2	8
**A**	1	4	1
**A**	1	4	2
**A**	1	4	4
**A**	1	4	8
**A**	1	8	1
**A**	1	8	2
**A**	1	8	4
**A**	1	8	8
**A**	8	1	1
**A**	8	1	2
**A**	8	1	4
**A**	8	1	8
**A**	8	2	1
**A**	8	2	2
**A**	8	2	4
**A**	8	2	8
**A**	8	4	1
**A**	8	4	2
**A**	8	4	4
**A**	8	4	8
**A**	8	8	1
**A**	8	8	2
**A**	8	8	4
**A**	8	8	8
**A**	2	1	8
**A**	2	8	1
**A**	4	1	8
**A**	4	2	8
**A**	4	8	1
**A**	4	8	2

Set	Dilution factor		
	hmAG1 (40 ng)	hmKO2 (10 ng)	tagBFP (200 ng)
**B**	1	1	16
**B**	1	4	16
**B**	1	16	1
**B**	1	16	4
**B**	1	16	16
**B**	4	1	16
**B**	4	16	1
**B**	16	1	1
**B**	16	1	4
**B**	16	1	16
**B**	16	4	1
**B**	16	16	1

Supplementary Table S4. List of oligoDNAs used in this study.

Type of DNA	Type of target	Primer name	Sequence (5' to 3')	Template for PCR
Forward	ORF	FwdEGFP	CACCGGTCGCCACCATGGGATCCGTGAGCAAGGGC	pEGFP-N1 (Clontech)
Reverse	ORF	RevEGFP	GCCCCGCAGAAGGTCTAGACCTACTTGTACAGCTCGTCCATGCCG	
Forward	ORF	FwdtagBFP	CACCGGTCGCCACCATGGGATCCAGCGAG	pSRT-tagBFP (this study)
Reverse	ORF	RevtagBFP	GCCCCGCAGAAGGTCTAGACTATCACTCGAGATGCATATGAGATC	
Forward	ORF	FwdhmAG1	CACCGGTCGCCACCATGGTGAGCGTGATCAAGCCCG	pFucci-S/G2/M Green (Amargaam)
Reverse	ORF	RevhmAG1	GCCCCGCAGAAGGTCTAGATTCACTTGGCCTGGCTGGGC	
Forward	ORF	FwdhmKO2	CACCGGTCGCCACCATGGTGAGTGTGATTAAACCAGAGATG	pFucci-G1 Orange (Amargaam)
Reverse	ORF	RevhmKO2	GCCCCGCAGAAGGTCTAGATTCAGGAATGAGCTACTGCATCTTCTACCTG	
Forward	ORF	FwdhdKRed	CACCGGTCGCCACCATGGTGAGCGTGATCGCCAAG	pNP-hdKeima-Red (Amargaam)
Reverse	ORF	RevhdKRed	GCCCCGCAGAAGGTCTAGATTCAGCCCAGCAGGCTGTGC	
Forward	ORF	FwdCBG68	CACCGGTCGCCACCATGGTGAAACGCGA	pCBG68-Control (Promega)
Reverse	ORF	RevCBG68	GCCCCGCAGAAGGTCTAGATTTACTAGCCGCCAGCTTTTTCG	
Reverse	ORF	RevM9	GCCCCGCAGAAGGTCTAGACTATCACTCGAGATGCATATGAGATC	
Forward	5' UTR	Fwd5UTR	CAGTGAATTGTAATACGACTCACTATAG	temp5UTR
Reverse	5' UTR	Rev5UTR	CATGGTGGCGACCGGTGTCTTATATTTCTTCTTACTC	
Template	5' UTR	temp5UTR	CAGTGAATTGTAATACGACTCACTATAGGGCGAATTAAGAGAGAAAAGAA GAGTAAGAAGAAATATAAGACACCGGTCGCCACCATG	
Forward	3' UTR	Fwd3UTR	TCTAGACCTTCTGCGGGGC	temp3UTR
Reverse	3' UTR	Rev3UTR	TTTTTTTTTTTTTTTTTTTTCCTACTCAGGCTTTATTCAAAGACCAAG	
Template	3' UTR	temp3UTR	TCTAGACCTTCTGCGGGGCTTGCCTTCTGGCCATGCCCTTCTTCTCTCCC TTGCACCTGTACCTCTTGGTCTTTGAATAAAGCCTGAGTAGG	
Forward	IVT	T7Fwd5UTR	CAGTGAATTGTAATACGACTCACTATAG	
Forward	IVT	T7FwdA	GCTAATACGACTCACTATAGGTCAGATCCGCTAGGATC	
Reverse	IVT	Rev120A	TTTTTTTTTTTTTTTTTTTTTTTTTTTTTTTTTTTTTTTTTTTTTTTTTT TTTTTTTTTTTTTTTTTTTTTTTTTTTTTTTTTTTTTTTTTTTTTTTTTT TTTTTTTTTTTTTTTTTTTTCCTACTCAGGCTTTATTCA	
Forward	vector	FwdMCS	GGGATCCCATGGTGTCGACCTGCAGCATATGAGCTCCTGAATTCGCCCTA TAGTGAGTCG	pGEM-T easy (Promega)
Reverse	vector	RevMCS	GGGAGATCTCATATGCATCTCGAGTGATAGTCTAGACAAGCTTGAGTATT CTATAGTGTCACC	
Insertion	vector	Code5UTR	AATTAAGAGAGAAAAGAAGAGTAAGAAGAAATATAAGAGCCAC	
Insertion	vector	Comp5UTR	CATGGTGGCTCTTATATTTCTTCTTACTCTTCTTTTCTCTCTT	
Insertion	vector	Code3UTR	CTAGACCTTCTGCGGGGCTTGCCTTCTGGCCATGCCCTTCTTCTCTCCCT TGCACCTGTACCTCTTGGTCTTTGAATAAAGCCTGAGTAGGA	
Insertion	vector	Comp3UTR	AGCTTCCTACTCAGGCTTTATTCAAAGACCAAGAGGTACAGGTGCAAGGG AGAGAAGAAGGGCATGGCCAGAAGGCAAGCCCCGCAGAAGGT	
Forward	cloning	CFwdtagRFP	GCCACCATGGGATCCGTGTCTAAGGGCGAAGAGC	pTagRFP-actin (Evarogen)
Reverse	cloning	CRevtagRFP	GCTCGAGATCTATTAAGTTTGTGCCCCAGT	
Forward	cloning	CFwdtagBFP	GCCACCATGGGATCCAGCGAGCTGATTAAGGAGAAC	pTagBFP-Tubulin (Evarogen)
Reverse	cloning	CRevtagBFP	ACTCGAGATCTGTGCCCCAGTTTGCTAG	
Forward	cloning	CFwdhmAG1	GCCACCATGGGATCCGTGAGCGTGATCAAGCCCG	pFucci-S/G2/M Green (Amargaam)
Reverse	cloning	CRevhmAG1	TATGAGATCTCTTGGCCTGGCTGGGC	
Forward	cloning	CFwdhmKO2	GCCACCATGGGATCCGTGAGTGTGATTAAACCAGAGATG	pFucci-G1 Orange (Amargaam)
Reverse	cloning	CRevhmKO2	TATGAGATCTGGAATGAGCTACTGCATCTTCTACCTG	
Forward	cloning	FwdM9	GAGATCCATGGGATCCAATCAGTCTTCAAATTTTGGAC	p4LambdaN22-3m EGFP-M9 (ref. 23)
Reverse	cloning	RevSV40	CTTTATTTGTAACCATTATAAGCTGC	

Supplementary Table S5. List of experimental conditions.

Experiment	Item	Cell	Transfected RNA	Analyzer
Translational efficiency	Fig. 2a, 2b, 3a, S3a, S3b, S3f, S3g, Table S1	HeLa, 293FT, MCF-7	miRNA-responsive EGFP (100 ng)<br>tagRFP (control, 100 ng)	Accuri C6
Two-cell separation	Fig. 2b–e, S2, S3c–e, S3h–j	HeLa, 293FT, MCF-7	miRNA-responsive EGFP (100 ng)<br>miRNA-responsive hmKO2 (125 ng)	Accuri C6
2-D cell separation	Fig. 3b–e, S5	HeLa, 293FT, MCF-7	miRNA-responsive hmAG1 (40 ng)<br>miRNA-responsive hmKO2 (10 ng)<br>miRNA-responsive tagBFP (200 ng)	FACSAria II
3-D cell separation	Fig. 3f, S7	HeLa, 293FT, MCF-7	miRNA-responsive hmAG1 (40 ng)<br>miRNA-responsive hmKO2 (10 ng)<br>miRNA-responsive tagBFP (200 ng)<br>miRNA-responsive hdKRed (250 ng)	FACSAria II
2-D separation space	Fig. S8, Table S3	HeLa	hmAG1 (control, 40 ng)<br>hmKO2 (control, 10 ng)<br>tagBFP (control, 200 ng)	FACSAria II
Cell identification with imaging cytometry	Fig. 4	HeLa, 293FT, MCF-7	miRNA-responsive hmAG1 (40 ng)<br>miRNA-responsive hmKO2 (10 ng)<br>miRNA-responsive tagBFP (200 ng)	IN Cell Analyzer 6000
